# Safety assessment of the indigenous probiotic strain *Lactiplantibacillus plantarum* subsp. *plantarum* Kita-3 using Sprague–Dawley rats as a model

**DOI:** 10.3934/microbiol.2022028

**Published:** 2022-11-01

**Authors:** Moh. A'inurrofiqin, Endang Sutriswati Rahayu, Dian Anggraini Suroto, Tyas Utami, Yunika Mayangsari

**Affiliations:** 1 Department of Food and Agricultural Product Technology, Faculty of Agricultural Technology, Universitas Gadjah Mada, Yogyakarta, Indonesia; 2 University Center of Excellence for Research and Application on Integrated Probiotic Industry, Universitas Gadjah Mada, Yogyakarta, Indonesia

**Keywords:** safety assessment, probiotic, bacterial translocation, *Lactiplantibacillus plantarum*

## Abstract

*Lactiplantibacillus plantarum* subsp. *plantarum* Kita-3 is a candidate probiotic from Halloumi cheese produced by Mazaraat Artisan Cheese, Yogyakarta, Indonesia. This study evaluated the safety of consuming a high dose of *L. plantarum* subsp. *plantarum* Kita-3 in Sprague-Dawley rats for 28 days. Eighteen male rats were randomly divided into three groups, such as the control group, the skim milk group, and the probiotic group. Feed intake and body weight were monitored, and blood samples, organs (kidneys, spleen, and liver), and the colon were dissected. Organ weight, hematological parameters, serum glutamic oxaloacetic transaminase (SGOT), and serum glutamic pyruvic transaminase (SGPT) concentrations, as well as intestinal morphology of the rats, were measured. Microbial analyses were carried out on the digesta, feces, blood, organs, and colon. The results showed that consumption of *L. plantarum* did not negatively affect general health, organ weight, hematological parameters, SGOT and SGPT activities, or intestinal morphology. The number of *L. plantarum* in the feces of rats increased significantly, indicating survival of the bacterium in the gastrointestinal tract. The bacteria in the blood, organs, and colon of all groups were identified using repetitive-polymerase chain reaction with the BOXA1R primers and further by 16S rRNA gene sequencing analysis, which revealed that they were not identical to *L. plantarum* subsp. *plantarum* Kita-3. Thus, this strain did not translocate to the blood or organs of rats. Therefore, *L. plantarum* subsp. *plantarum* Kita-3 is likely to be safe for human consumption.

## 1. Introduction

Probiotics are live microorganisms that confer a health benefit to the host when administered in adequate amounts [1]. Probiotic organisms commonly belong to the genera *Lactobacillus* and *Bifidobacterium*, which are lactic acid bacteria (LAB) [2]. Many probiotics have their origins in dairy products and fermented foods. LAB have been consumed for many years as components of these foods without any apparent adverse effects on consumer health. LAB has been accepted as safe in the absence of scientific criteria because they are microflora typically found in the intestinal tract and have a long history of application without adverse effects [3].

LAB can be classified as a probiotic if it provides health benefits, survives in gastric acid and bile salts, adheres to the intestinal mucosa surface, and has antimicrobial properties against pathogens [4]. *Lactiplantibacillus plantarum* (formerly *Lactobacillus plantarum*) is a common LAB found in Indonesian fermented foods and dairy products [5]. *Lactiplantibacillus plantarum* subsp. *plantarum* Kita-3 is a potential indigenous probiotic strain isolated from Halloumi cheese produced by Mazaraat Artisan Cheese, Yogyakarta, Indonesia. This bacterium met the basic requirements for a probiotic and has some functional properties. *L. plantarum* subsp. *plantarum* Kita-3 is highly resistant to simulated gastric juice and bile salts. This strain effectively adheres to human epithelial cells and protects the gut against pathogenic bacteria, such as *Salmonella typhi*, *Escherichia coli*, *Shigella dysenteriae*, and *Staphylococcus aureus* because it has antibacterial activities [6]. The use of *L. plantarum* subsp. *plantarum* Kita-3 is part of an effort to commercialize local strains to compete in the global market.

Although *Lactobacillus* sp. has received Generally Recognized as Safe status [7], the safety of this bacterium must be further evaluated. The safety of new probiotic strains needs to be studied as probiotic bacteria are strain-specific [8]. Several significant risks may potentially occur when probiotics are supplemented in humans, such as the risk of infection, impaired metabolism, impaired immunity, and transfer of antibiotic-resistant genes [9]. Several studies have reported cases of bacteremia associated with the genus *Lactobacillus* [10]. Therefore, before using *L. plantarum* subsp. *plantarum* Kita-3 for long-term human consumption, its safety features, and its possible use as a probiotic need to be researched before further implementation in the industrial-scale food system.

A safety assessment of a probiotic strain can be applied using a rat model to confirm the possibility of the bacterium causing infection. A safety study evaluated *Lactobacillus plantarum* Dad-13 by introducing this strain orally at a high dose of 10^11^ CFU/rat/day for 28 days [11]. The safety assessment was carried out by monitoring rat viability, feed intake, body weight, organ weight, intestinal morphology, and performing blood biochemical and blood and organ bacterial analyses of the rats. The results showed no significant effects due to the treatment; thus, the probiotic was judged to be safe for consumption.

Molecular techniques that are faster and more accurate have been developed to identify bacteria with several advantages, such as including bacteria that have not been identified [12]. Bacterial DNA amplification using repetitive-polymerase chain reaction (rep-PCR) analysis has been acknowledged as a straightforward inexpensive PCR approach suitable for characterizing LAB, such as *Lactobacillus*, to the strain level when compared to other PCR techniques, such as pulsed-field gel electrophoresis and amplified fragment length polymorphism [13]. A previous safety assessment study for the probiotic strain *Lactobacillus plantarum* Mut-7 identified 26 bacterial isolates from the blood and organs of rats, which were identified using rep-PCR [14].

Additionally, the 16S rRNA gene is frequently used in DNA sequencing analysis. 16S rRNA is used as a molecular marker because it is widely distributed; therefore, it can be used as a universal primer for all bacterial groups. If the similarity of the 16S rRNA gene sequence is <97.5%, the bacteria is considered a new species because the two bacteria are unlikely to have DNA similarities of more than 60%–70% [15]. In addition, 16S rRNA gene sequence data from the bacterial isolates were compared with 16S rRNA gene sequence data from the reference bacterial species taken from the GeneBank database.

This study was carried out to evaluate the safety of *L. plantarum* subsp. *plantarum* Kita-3 in a Sprague-Dawley rat model by administering this strain orally at a high dose of 10^11^ CFU/rat/day for 28 days. Several variables were examined, including feed intake, body weight, organ weight, hematological status, and biochemical markers, such as serum glutamic oxaloacetic transaminase (SGOT) and serum glutamic pyruvic transaminase (SGPT), gastrointestinal tract morphology, and bacterial analyses of the digesta, feces, blood, organs (kidneys, spleen, and liver) and colon of the rats. The possibility that *L. plantarum* subsp. *plantarum* Kita-3 was translocated to the blood and organs of rats was also examined.

## 2. Materials and methods

### 2.1. Bacterial strain and cell production

*L. plantarum* subsp. *plantarum* Kita-3 was collected from the Food and Nutrition Culture Collection, Center for Food and Nutrition Studies, Universitas Gadjah Mada, Yogyakarta, Indonesia. The biomass was produced by inoculating 0.1 mL of *L. plantarum* subsp. *plantarum* Kita-3 into 10 mL of de Man Rogosa Sharpe (MRS) broth (Merck, Darmstadt, Germany). Then, we further inoculated that into 1 L of MRS broth and incubated at 37 °C for 24 h. The culture was cold centrifuged for 10 min at 3,500 rpm and the pellet was resuspended in 20 mL of 10% skim milk solution at a concentration of 10^11^ CFU/mL. Then, the biomass was enumerated and stored at −20 °C.

### 2.2. Experimental design

In this study, rats were used as the research subjects following the Declaration of Helsinki guidelines. The Ethical Clearance Committee, Integrated Research and Testing Laboratory, Universitas Gadjah Mada, Yogyakarta, Indonesia, approved the study protocol, according to the ethical clearance certificate numbered 00048/04/LPPT/X/2021 and dated October 29, 2021. Eighteen male Sprague–Dawley rats (age, 8 weeks; body weight, 200 ± 20 g) were housed individually in stainless steel cages with adequate air circulation, a 12 h/12 h light-dark cycle, and controlled room conditions (temperature of 22 °C–25 °C and relative humidity of 60%). The rats were fed the AIN-93M diet (Center for Food and Nutrition Studies, Yogyakarta, Indonesia) and provided water *ad libitum* based on the method in Reeves *et al*. [16] with modifications. The AIN-93M diet composition included 62.07% corn starch, 14% casein, 10% sucrose, 4% soybean oil, 5% fiber, 3.5% mineral mix, 1% vitamin mix, 0.18% L-cystine, and 0.25% choline bitartrate.

The rats were conditioned under normal, healthy conditions. The force-feeding intervention was administered to the rats after they had completed a 1-week adaptation period to the experimental conditions with the AIN-93M diet. The rats were randomly divided into three groups of six rats per group. The first group (P.1) was fed the AIN-93M diet for 28 days. The second group (P.2) was fed the AIN-93M diet and 1 mL of 10% skim milk solution for 28 days. The third group (P.3) was fed the AIN-93M diet and 1 mL of 10^11^ CFU *L. plantarum* subsp. *plantarum* Kita-3 in a 10% skim milk solution for 28 days.

The activity and behavior of each rat were observed and recorded daily. The feed intake and body weight of the rats were recorded every day. All rats were euthanized with ketamine on day 29, and blood samples were taken. The anatomy of the organs of each rat was checked and recorded, and the organ weight index was calculated. Subsequently, the rat organs (kidneys, spleen, and liver) and intestinal tract were aseptically collected in a refrigerated box, and formalin was added for further analyses.

### 2.3. General health and hematological and biochemical analyses

General health analysis was carried out by evaluating feed intake and body weight according to Shu *et al*. [17]. The feed intake of the rats was determined by calculating the difference between feed weight and the remaining feed (g). The change in body weight was determined by calculating the difference between the last and initial body weights. The kidneys, spleen, and liver were weighed and the organ weight index was calculated using the formula in Zhou *et al*. [18]: [Organ weight (g)/Last body weight (g) × 100].

Blood samples were obtained through the orbital sinus of the eye using sterile microhematocrit tubes and saved aseptically in an EDTA tube. Hematological status was determined automatically using the KX-21 Hematology Analyzer (Sysmex, Kobe, Japan) with fresh blood based on the principle of flow cytometry [18].

The blood samples were analyzed for SGOT and SGPT as biochemical markers. Enzyme reagents and reagent starters were used to react with the serum, which was collected by centrifugation. SGOT and SGPT were measured using the DyaSis kit, and absorbance was measured at 340 nm using the MicroLab 300 spectrophotometer (ELITech, Braeside, Australia) [19].

### 2.4. Gut histological analysis

The histology of the intestinal tract was examined to evaluate changes in mucosal integrity due to the treatments. The morphology of the intestinal tract (ileum, caecum, and colon) was qualitatively identified using an Eclipse E100 microscope (Nikon, Tokyo, Japan). A quantitative analysis was conducted by examining the height of the villi, the height of the epithelium, and the thickness of the mucosa. Hematoxylin and eosin (H&E) staining was used on the tissue samples based on the method of Zhou *et al*. [20] with modifications. The tissue samples were immersed for 48 h in 10% neutral buffered formalin. Then, the tissue samples were cut to 0.3–0.5 mm thickness, placed in a basket, dehydrated, and the air was removed. Paraffin blocks were molded and the tissue blocks were cut into 3–4 µm lengths. The tissue blocks were stained with H&E solution.

### 2.5. Bacterial analyses of the digesta, feces, blood, organs, and the colon

One g each of the digesta, feces, and colon samples were homogenized and serially diluted with 9 mL of a 0.9% sodium chloride solution. A 0.1 ml aliquot of sample in MRS agar (Oxoid, Basingstoke, UK) and *Lactiplantibacillus plantarum* Selective Media (LPSM) was spread on plates for each dilution. The plates were incubated at 37 °C for 48 h. The colonies were enumerated using the Quebec Darkfield Colony Counter (Reichert, NY, USA) based on the method of Bujalance *et al*. [21] with modifications.

To evaluate the possibility of translocation of the bacteria to the blood, organs (kidneys, spleen, and liver), and colon of the rats, a 0.1 mL of sample from each organ was enriched in a test tube containing MRS broth and incubated at 37 °C for 24 h. In addition, a bacterial adhesion test was carried out using the method of Saxami *et al*. with modifications [22]. A 3 cm long longitudinal cut was made aseptically in the colon. After removing the intestinal fluid, the tissue sample was washed with sterile sodium chloride solution and vortex mixed to break the bacterial clumps and remove loosely attached bacteria. It was then swabbed with a sterile cotton swab and introduced into a test tube containing MRS broth and incubated at 37 °C for 24 h.

Furthermore, each sample was inoculated on MRS agar and LPSM plates using the streak method and incubated at 37 °C for 48 h until a single colony was obtained. The colonies that appeared on the MRS agar and LPSM plates were thought to be LAB and *L. plantarum,* respectively, so they were analyzed for colony and cell morphology, Gram staining, and catalase based on the methods of Zhou *et al*. [18] with modifications. The colonies were analyzed by visually observing the shape, color, appearance, and edges. Cell morphology was determined using the Eclipse E100 microscope (Nikon).

The catalase test was carried out by dripping 3% hydrogen peroxide (H_2_O_2_) on a glass slide containing a smear of the culture; then, the culture was mixed slowly with a loop. A positive result was indicated by the formation of oxygen bubbles, while no bubbles indicated a negative result [23].

### 2.6. Bacterial genome DNA isolation

Bacterial genomic DNA was isolated from the rats' blood, organs, and colon using the Presto™ Mini gDNA Bacteria Kit (Geneaid, New Taipei City, Taiwan) based on the manufacturer's protocol [24]. (1) Sample preparation: The bacterial cells (up to 1 × 10^9^) were transferred to a 1.5 mL microcentrifuge tube, which was centrifuged at 14,000–16,000 × g for 1 min, and the supernatant was discarded. A 200 µL aliquot of the Gram + Buffer was transferred per sample to a 15 mL centrifuge tube, Lysozyme (0.8 mg/200 µL) was added and vortexed to fully dissolve the Lysozyme. The pellet was resuspended and 200 µL of Gram + Buffer (ensure Lysozyme was added) was added to the sample in the 1.5 mL microcentrifuge tube and incubated for 30 min at 37 °C. The tube was inverted every 10 min during the incubation. A 20 µL aliquot of Proteinase K was added, vortexed, and incubated for at least 10 min at 60 °C. The tube was inverted every 3 min during the incubation; (2) Lysis: A 200 µL aliquot of GB Buffer was transferred to the sample, vortex mixed for 10 s, and incubated for at least 10 min at 70 °C to make sure the sample lysed. The tube was inverted every 3 min throughout the incubation. The Elution Buffer (200 µL per sample) was pre-heated to 70 °C; (3) DNA binding: Transfer 200 µL of absolute ethanol to the sample lysate and vigorously shake the mixture, to break up the precipitate. The mixture was transferred (along with any insoluble precipitate) to a GD column with a 2 mL collection tube. Centrifuge the mixture at 14,000–16,000 × g for 2 min and remove the 2 mL collection tube holding the flow-through. Add a new 2 mL collection tube to the GD column ; (4) Wash: Transfer 400 µL of W1 Buffer to the GD column, centrifuge at 14,000–16,000 × g for 30 s; remove the flow-through, put the GD column back on the 2 mL collection tube, transfer 600 µL of Wash Buffer (ensure ethanol was added) to the GD column, centrifuge at 14,000–16,000 × g for 30 s; remove the flow-through, put the GD column back with the 2 mL collection tube and centrifuge again for 3 min at 14,000–16,000 × g to dry the column matrix; (5) Elution: The dried GD column was filled and attached to a clean 1.5 mL microcentrifuge tube; 100 µL of pre-heated Elution Buffer was transferred into the center of the column matrix, allowed to stand for at least 3 min to ensure that the Elution Buffer was fully absorbed and centrifuged at 14,000–16,000 × g for 30 s to elute the purified DNA. Then, the isolated DNA was stored at −20 °C.

### 2.7. Repetitive-polymerase chain reaction amplification

The rep-PCR method was used to identify the genetic diversity of the bacterial isolates detected in the blood, organs, and colon. The PCR-mix Ready to Go (RTG) was employed as the starting material for the PCR amplification procedure. One µL of DNA, 1 µL of the BOXA1R primer (5′CTACGGCAAGGCAAGGCGACGCTGACGCTGACG-3′), and 23 µL of nuclease-free water were added to each PCR-mix RTG reaction ampoule. The PCR amplification was carried out using the Thermal Cycler TC1000-G (DLAB Scientific, Beijing, China) comprised of several steps based on the method of de Bruijn [25] with modifications. (1) Initial denaturation (94 °C for 4 min) in one cycle, (2) 30 cycles of denaturation (92 °C for 1 min), annealing (50 °C for 1 min) and extension (68 °C for 8 min) and (3) one final cycle (65 °C for 10 min). The PCR reaction products were visualized by electrophoresis with ethidium bromide staining on a 2% agarose gel soaked with 100 mL of 0.5 × TBE at 100 V for 30 min. The DNA from *L. plantarum* subsp. *plantarum* Kita-3 was used as the reference and was compared to the bacterial isolates from the blood, organs, and colon to obtain fingerprints that were used to develop the similarity dendrogram.

### 2.8. 16S rRNA gene amplification and sequencing analysis

One µL of DNA, 1 µL each of the 27F and 1492R primers (AGAGTTTGATCCTGGCTCAG and GGTTACTTGTTACGACTT), and 22 µL of nuclease-free water were added to each PCR-mix RTG ampoule. There were multiple phases involved in the PCR amplification process using the Thermal Cycler TC1000-G (DLAB Scientific): (1) Initial denaturation (96 °C for 4 min) in one cycle, (2) 35 cycles of denaturation (94 °C for 1 min), annealing (52 °C for 1.5 min) and extension (65 °C for 8 min), (3) one final cycle (68 °C for 10 min) and (4) cooling (12 °C for 10 min), followed by electrophoresis of the DNA fragments.

The next step was 16S rRNA gene sequencing of *L. plantarum* subsp. *plantarum* Kita-3 and the suspected *L. plantarum* from the blood, organs, and colon. DNA sequencing was carried out by the FIRST BASE DNA Sequencing Service, Genetika Science, Tangerang, Indonesia. DNA Baser software was used to read the outcomes of the subsequent DNA sequences. Additionally, the Alignment program in BioEdit software was chosen to complete the matching procedure. The Basic Local Alignment Search Tool approach was used to detect similar results for the DNA sequences that were comparable to or identical to the DNA sequence data for genes from the GenBank of the National Center for Biotechnology Information (NCBI) database [14]. The DNA sequence was applied as reference data to design the phylogenetic tree. The NCBI GenBank worldwide database was used to retrieve the DNA sequence data. The DNA sequences of a few chosen bacteria were saved as reference isolates for additional genetic research using MEGA XI software [26].

### 2.9. Statistical analysis

The data are expressed as mean ± standard deviation. Statistical analysis was carried out with a one-way analysis of variance and Duncan's multiple range test to detect differences A *p*-value of <0.05 was considered significant using SPSS Statistics 22 software (IBM Corp., Armonk, NY, USA).

## 3. Results and discussion

### 3.1. Health indicators and hematological and biochemical parameters

Force feeding a high dose of *L. plantarum* subsp. *plantarum* Kita-3 did not significantly affect the feed intake or body weight of any rat group. The organ weight indices of the groups are displayed in Table 1. The mean organ weight index was not different among the groups (*p* > 0.05). Administering the probiotic for 28 days did not cause a bacterial infection in the organs of the rats. In addition, all of the rats survived until day 28. Their physiological parameters were constant, and no negative consequences were detected throughout the probiotic consumption period.

**Table 1.** The organ weight index per group.

Group	Organ (g)		
	Kidneys	Spleen	Liver
P.1	0.81 ± 0.05	0.69 ± 0.10	2.73 ± 0.30
P.2	0.79 ± 0.03	0.58 ± 0.11	2.47 ± 0.21
P.3	0.77 ± 0.08	0.56 ± 0.17	2.47 ± 0.33

Note: No significant differences were detected between the groups (*p* > 0.05). P.1 = control group, P.2 = skim milk group and P.3 = probiotic group.

The hematological analysis revealed no significant difference in any of the hematological parameters in any of the groups of rats (*p* > 0.05), as shown in Table 2. Hematological parameters, such as the white blood cell count, are effective indicators for detecting a bacterial infection [27]. Treatment with a high dose of *L. plantarum* subsp. *plantarum* Kita-3 did not increase the leukocyte profile or total leukocytes in the blood of the rats. Significant increases in white blood cell counts would indicate an immune reaction to a pathogen. Monocytes play an essential role in preventing intestinal pathogens by releasing macrophages, while neutrophils are responsible for phagocytosing microbes and producing antimicrobial factors [28]. According to these results, a high dose of *L. plantarum* subsp. *plantarum* Kita-3 for 28 days did not cause a blood bacterial infection.

**Table 2.** Blood hematology test results by group.

Parameter	Group			Ref
	P.1	P.2	P.3	
WBC (×10^3^/µL)	15.08 ± 3.06	12.63 ± 1.45	13.82 ± 4.86	5.30
RBC (×10^6^/µL)	7.98 ± 1.02	7.40 ± 0.60	7.40 ± 0.65	8.20
HGB (g/dl)	14.70 ± 1.81	14.15 ± 1.66	12.77 ± 1.08	14.60
HCT (%)	48.80 ± 6.62	47.62 ± 5.37	43.27 ± 4.38	44.60
MCV (fl)	61.17 ± 1.17	58.58 ± 2.12	58.52 ± 3.41	54.40
MCH (pg)	18.50 ± 1.74	18.23 ± 1.87	17.28 ± 0.69	17.78
MCHC (g/dl)	30.25 ± 2.70	28.15 ± 2.57	29.53 ± 0.72	32.72
PLT (×10^3^/µL)	1006.33 ± 339.94	1108.33 ± 314.41	1028.33 ± 191.63	870.00
LYM (%)	72.05 ± 19.46	72.65 ± 13.33	77.10 ± 12.82	76.80
LYM# (×10^3^/µL)	10.77 ± 2.89	10.22 ± 3.38	11.08 ± 5.20	4.16
NEUT (%)	27.95 ± 19.46	22.78 ± 12.63	22.90 ± 12.82	14.10
NEUT# (×10^3^/µL)	4.32 ± 2.97	3.40 ± 2.15	2.73 ± 1.11	1.03
SGOT (U/L)	170.32^b^ ± 26.55	161.70^b^ ± 24.88	118.47^a^ ± 30.92	89.00
SGPT (U/L)	74.15 ± 15.33	73.03 ± 9.67	72.07 ± 18.06	68.00

Note: Different superscripts in the same row indicate a significant difference (*p* < 0.05). P.1 = control group, P.2 = skim milk group and P.3 = probiotic group. Ref = reference [19]; WBC = leukocytes; RBC = red blood cells; HGB = hemoglobin; HCT = hematocrit; MCV = mean corpuscular volume average; MCH = mean corpuscular hemoglobin; MCHC = mean corpuscular hemoglobin concentration; PLT = platelet count; LYM = lymphocytes; NEUT = neutrophils; SGOT = serum glutamic oxaloacetic transaminase; SGPT = serum glutamic pyruvic transaminase.

The administration of a high dose of *L. plantarum* subsp. *plantarum* Kita-3 had no significant effect on the biochemical parameters, such as SGOT and SGPT activities, among the groups (*p* > 0.05). The levels of SGOT and SGPT were higher in all groups than the normal reference values, indicating hepatocyte injury. The high levels of SGOT and SGPT could also be associated with organ damage and chronic diseases, including infection [29]. It is reasonable to assume that the consumption of a high dose of *L. plantarum* subsp. *plantarum* Kita-3 was not the cause of the increase in SGOT and SGPT levels. A previous study showed SGOT activity of 184.3 U/l and 199.8 U/l for control and treatment groups that consumed 10^10^ CFU/day/rat *Lactobacillus salivarius* CECT5713, respectively [30]. These levels are higher than the SGOT levels in this study. SGOT and SGPT are markers of physiological change. They exist not only in the liver but also in other organs [29]. High activity can be an indicator of organ damage. Moreover, high levels of SGOT and SGPT have been associated with chronic diseases, including infections [29]. The finding that the SGOT and SGPT activities did not increase indicates that the administration of the high dose of *L. plantarum* subsp. *plantarum* Kita-3 for 28 days did not cause any physiological changes in the rats.

### 3.2. Gut histology analysis

**Figure 1.**
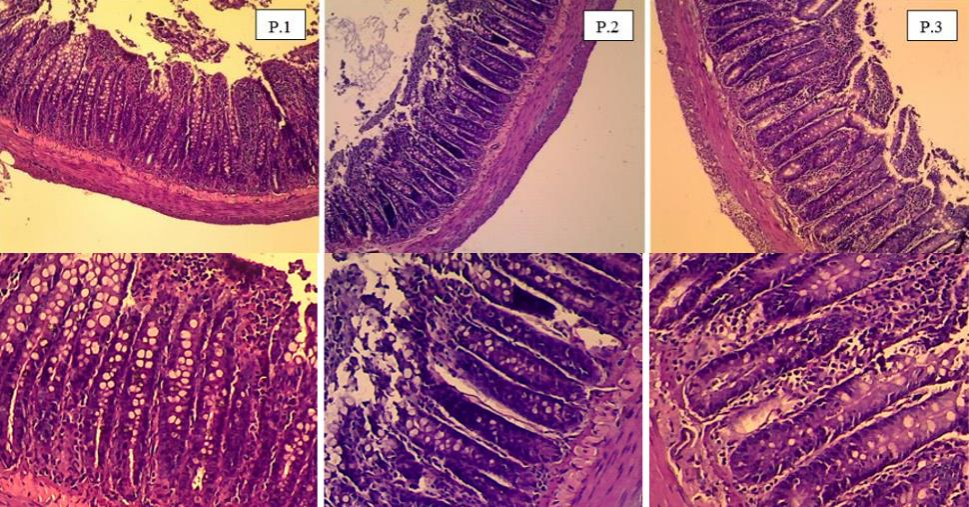
Microscopic appearance of the ileum (hematoxylin and eosin stain). P.1 = control group; P.2 = skim milk group; P.3 = probiotic group.

The histological examination of the gut revealed no inflammation following consumption of *L. plantarum* subsp. *plantarum* Kita-3, which would have caused a continued infection. Figure 1 shows that the gut morphology was normal and no erosion was detected on the mucosal surface. Mucosal erosion occurs when the tissue thins on the outside of the mucosa [31]. This result was supported by quantitative analysis of the gut morphology (Table 3). No significant differences in the height of the villi, the height of the epithelium, or the thickness of the mucosa were observed among the groups (*p* > 0.05).

**Table 3.** Quantitative analysis of the intestinal morphology by group.

Intestinal tract	Parameter (µm)	Group		
		P.1	P.2	P.3
Ileum	Villous height	235.01 ± 18.54	275.06 ± 74.20	307.06 ± 77.30
	Epithelial height	19.12 ± 4.03	21.32 ± 4.26	19.80 ± 5.79
	Mucosal thickness	21.67 ± 5,72	21.50 ± 4,68	20.67 ± 5,32
Caecum	Epithelial height	16.81 ± 2.22	16.77 ± 2.91	17.97 ± 2.60
	Mucosal thickness	256.48 ± 44.49	232.09 ± 10.19	230.70 ± 45.06
Colon	Epithelial height	20.53 ± 4.39	24.09 ± 5.76	19.73 ± 2.25
	Mucosal thickness	202.50 ± 40.97	200.76 ± 22.46	235.05 ± 17.12

Note: The treatments did not change any of the parameters between the groups (*p* > 0.05). P.1 = control group; P.2 = skim milk group; P.3 = probiotic group.

The results showed no significant differences in the ileum, cecum, or colon of the probiotic group compared with the control and skim milk groups at the end of the treatment period. These results indicate that administering a high dose of *L. plantarum* subsp. *plantarum* Kita-3 for 28 days did not change the gut structure or interfere with the integrity of the intestinal tract. Inflammation due to changes in gut structure is characterized by the appearance of reddish spots, shortening or elongation of the intestinal villi, and enlargement of globules in the ileal mucosa [32]. In addition, inflammation also occurs due to erosion of the mucosa caused by the invasion of pathogenic bacteria [31]. A previous study revealed that administering *Lactobacillus rhamnosus* HN001, *Lactobacillus acidophilus* HN017, and *Bifidobacterium lactis* HN019 to rats did not have any adverse effects on intestinal integrity [18]. Intestinal and gut barriers are the first defense mechanisms in preventing pathogen invasion of the blood [33]. Bacteria commonly pass through the epithelial cells in the intestine and interact with immune cells under normal conditions, but if the mucosa and epithelial cells are damaged, immune cell interactions are excessive, causing inflammation in the intestinal tract and further triggering sepsis when the bacteria translocate to the blood [34]. Bacterial translocation is caused by impaired intestinal integrity and permeability, therefore the bacteria can penetrate the blood circulation and organs, making the host susceptible to disease [35].

### 3.3. Populations of lactic acid bacteria and Lactiplantibacillus plantarum in the digesta, feces, and colon of rats

The digesta and feces of the rats contained *L. plantarum* subsp. *plantarum* Kita-3 after consuming it for 28 days, which increased the probiotic group LAB count to 10^8^ CFU/mL (Table 4). This finding is consistent with Rahayu *et al*. [11], in which the consumption of *L. plantarum* subsp. *plantarum* Dad-13 resulted in up to 10^8^ CFU/mL of LAB in the digesta and feces of rats. The numbers of LAB in the digesta and feces were not much different, indicating that *L. plantarum* subsp. *plantarum* Kita-3 survives in the feces of rats.

**Table 4.** The populations of LAB and *L. plantarum* in the digesta, feces, and colon of rats using MRS agar and LPSM.

Group	LAB (log CFU/mL)						*L. plantarum* (log CFU/mL)					
	Digesta			Feces			Digesta			Feces		
	Mean	Min	Max	Mean	Min	Max	Mean	Min	Max	Mean	Min	Max
P.1	5.03	4.59	6.03	5.32	4.62	6.23	nd	nd	nd	nd	nd	nd
P.2	5.57	4.88	6.38	5.68	4.64	6.96	nd	nd	nd	nd	nd	nd
P.3	8.53	8.04	8.91	8.25	7.93	8.55	8.34	7.55	9.17	8.03	7.75	8.39

Note: nd = not detected. P.1 = control group; P.2 = skim milk group; P.3 = probiotic group.

The populations of *L. plantarum* in the digesta and feces of the control and skim milk group rats were very low and consumption of a high dose of *L. plantarum* subsp. *plantarum* Kita-3 for 28 days markedly increased their numbers. *L. plantarum* was detected in the probiotic group with an average growth of 10^7^ CFU/mL, which promoted the growth of microflora in the digesta and feces. These results suggest that *L. plantarum* subsp. *plantarum* Kita-3 survived and grew in the gastrointestinal tract of the rats. Ratna *et al*. [6] reported that *L. plantarum* subsp. *plantarum* Kita-3 survives in gastric juice with a pH of 2, survives in 0.3% bile salt solution and inhibits pathogenic bacteria, such as *Salmonella typhi, Escherichia coli, Shigella dysenteriae*, and *Staphylococcus aureu*s. In addition, the results of the bacterial analysis in the colon showed that the population in the probiotic group was higher than that in the control and skim milk groups (Table 5). The population of LAB and *L. plantarum* in the colon indicated that *L. plantarum* subsp. *plantarum* Kita-3 can adhere well to the mucosal surface of the gastrointestinal tract. This adhesion property is an important criterion for a probiotic [36].

**Table 5.** The populations of LAB and *L. plantarum* in the colon of rats using MRS agar and LPSM.

Group	The bacterial population in the colon (log CFU/mL)					
	LAB (MRS agar)			*L. plantarum* (LPSM)		
	Mean	Min	Max	Mean	Min	Max
P.1	5.62	5.40	5.95	4.24	4.06	4.85
P.2	5.64	5.59	5.76	4.12	3.98	4.31
P.3	7.30	6.70	7.69	7.22	6.36	7.55

Note: P.1 = control group; P.2 = skim milk group; P.3 = probiotic group.

### 3.4. Bacterial isolation from the blood, organs, and colon of the rats

The results of bacterial analyses of the blood, organs (kidneys, spleen, and liver), and colon of the rats revealed that all samples contained bacteria that grew on MRS agar (Table 6). Bacterial isolates from the blood, organs, and colon were suspected to be LAB. However, it was interesting that LAB was found in the control rats' blood, organs, and colon. The blood, organs, and colon may have already contained the bacteria.

**Table 6.** The number of positively detected bacterial isolates from the rats' blood, organs, and colon.

Isolate source	MRS agar (LAB)			LPSM (*L. plantarum*)		
	P.1	P.2	P.3	P.1	P.2	P.3
Blood	3/6	2/6	1/6	2/6	1/6	1/6
Kidneys	3/6	2/6	2/6	1/6	2/6	2/6
The spleen	2/6	1/6	1/6	2/6	1/6	1/6
Liver	3/6	2/6	2/6	1/6	2/6	1/6
Colon	6/6	6/6	6/6	4/6	3/6	6/6

Note: a/b = number of detected bacterial isolates/number of examined tissue samples. P.1 = control group; P.2 = skim milk group; P.3 = probiotic group.

The bacterial analysis using LPSM in all groups revealed bacterial colonies that were suspected to be *L. plantarum*. Based on this result, the probiotic group had more bacteria than the other groups. However, it has not been confirmed that this was related to the administration of *L. plantarum* subsp. *plantarum* Kita-3 considering that bacteria were also found on LPSM in the control and skim milk groups. In this study, bacterial translocation could not be determined because the intestinal tract was not inflamed. This agrees with the finding that *Lactobacillus* cannot translocate, but translocation occurs when the intestinal mucosa is disturbed or the immune system is unable to inhibit the invasion of a pathogen, resulting in translocation that triggers infection [8]. The bacterial translocation that causes systemic infection of organs occurs due to injury or an inflammatory reaction that causes changes in intestinal permeability [37]. A positive blood or organ culture of the treated rats did not prove that the probiotic was to blame for the bacterial translocation because it was also detected in untreated rats [38]. Bujalance *et al*. [21] revealed that several bacteria, such as *Escherichia coli*, *Streptococcus thermophilus*, *Salmonella enterica, Lactobacillus casei*, and *Lactobacillus fermentum*, can grow on LPSM in addition to *L. plantarum*.

These results agree with a previous study reporting that LAB was found in a kidney sample after translocation tests for *Lactobacillus rhamnosus* HN001 and *Bifidobacterium lactis* in rat organs; however, molecular analysis revealed that the bacteria did not originate from the probiotic [20]. Other studies on the safety of probiotics in rats also detected LAB in organs [30],[20]. Another study stated that translocation cases in the control and treatment groups are not indicated due to the administration of probiotics [18]. In addition, *L. plantarum* cannot pass through the gastrointestinal membrane barrier [39]. Therefore, further studies are necessary to investigate the bacteria in the rats' blood, organs, and colon and their genetic relationship with *L. plantarum* subsp. *plantarum* Kita-3.

Thirty bacterial isolates taken from the rats' blood, organs, and colon grew on LPSM. However, it was not confirmed whether those bacteria originated from consuming *L. plantarum* subsp. *plantarum* Kita-3, as these bacteria grew on LPSM in the control group. Fifteen isolates were Gram-positive rod cells, and six were Gram-positive cocci. There were also nine Gram-negative bacteria indicative of non-LAB. Gram-positive bacteria with short rod-shaped cells that ferment sorbitol and grow at 10 °C–45 °C were *L. plantarum* [40].

Furthermore, four of the 15 Gram-positive rod cells produced catalase, which is not a LAB characteristic. The positive reaction on the catalase test was indicated by the formation of oxygen bubbles showing that the bacteria produced catalase, which converts hydrogen peroxide to water and oxygen, but LAB do not produce catalase [23]. Thus, 11 isolates from the rats' blood, organs, and colon were further analyzed at the molecular level, as shown in Table 7.

**Table 7.** The bacterial isolates from rats' blood, organs, and colon were further identified at the molecular level.

Isolate source	Group		
	P.1	P.2	P.3
Blood	NI	1 (DRS5)	1 (DRK6)
Kidneys	1 (GJC2)	1 (GJS4)	NI
The spleen	1 (LPC1)	NI	1 (LPK6)
Liver	1 (LVC1)	1 (LVS1)	NI
Colon	1 (KLC3)	1 (KLS4)	1 (KLK5)

Note: P.1 = control group; P.2 = skim milk group; P.3 = probiotic group. NI = None Isolated. The text in brackets is the isolate code (DRS5 = isolate from blood in P.2, DRK6 = isolate from blood in P.3, GJC2 = isolate from kidneys in P.1, GJS4 = isolate from kidneys in P.2, LPC1 = isolate from spleen in P.1, LPK6 = isolate from spleen in P.3, LVC1 = isolate from liver in P.1, LVS1 = isolate from liver in P.2, KLC3 = isolate from colon in P.1, KLS4 = isolate from colon in P.2 and KLK5 = isolate from colon in P.3).

However, one study reported that *E. faecalis* can use sorbitol as a source of energy and produces lactic acid [41]. Moreover, *E. faecalis* is resistant to the antibiotic ciprofloxacin contained in LPSM [42]. The bacteria found in the blood, organs, and colon of the rats may not have been the result of consuming a high dose of *L. plantarum* subsp. *plantarum* Kita-3. It could be other bacteria, such as *E. faecalis*.

### 3.5. Bacterial identification based on rep-PCR using the BOXA1R primer

Further analyses were conducted using genotypic and molecular methods to further identify the bacteria isolated from the rats' blood, organs, and colon. Thus, the methods could be used to distinguish the strain of *L. plantarum* subsp. *plantarum* Kita-3 from the other isolates obtained from the rats' blood, organs, and colon. Rep-PCR is a fingerprinting method used to differentiate between LAB strains, such as *Lactobacillus johnsonii* [13] and *Bifidobacterium,* using the BOXA1R primer [43]. The bands produced following rep-PCR amplification with the BOXA1R primer show complex patterns to discriminate at the sub-species level [44].

**Figure 2.**
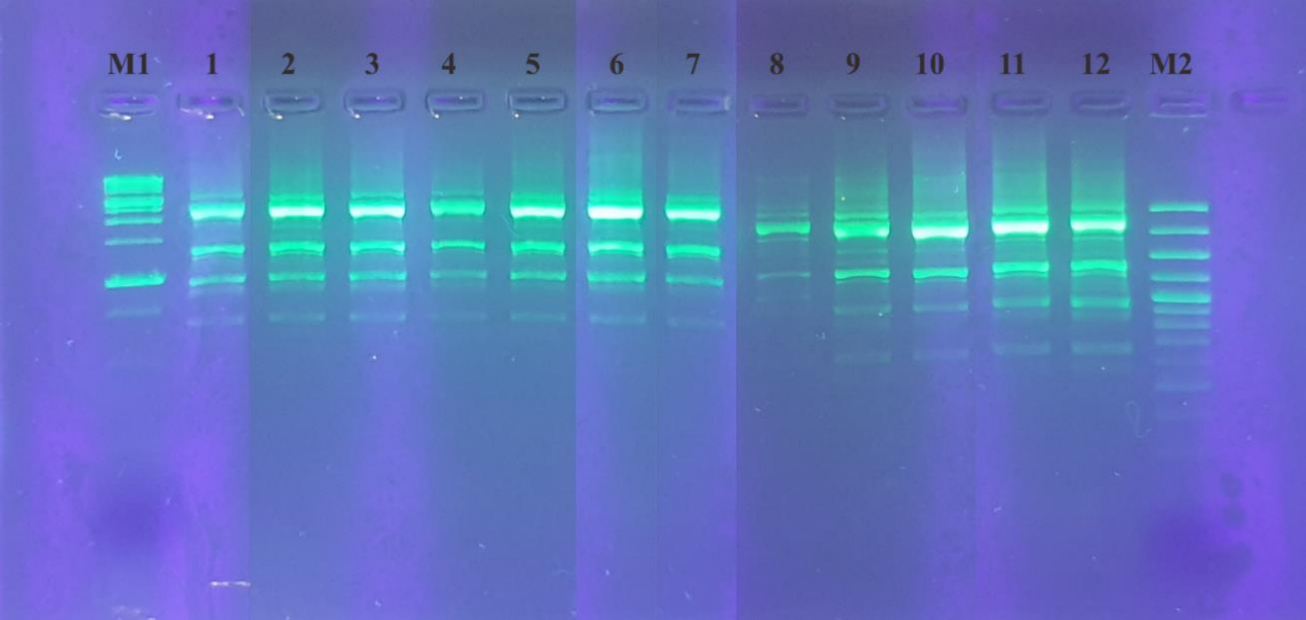
The agarose gel electrophoresis result from rep-PCR using the BOXA1R primer for the 12 bacterial isolates. M1 = 1 kb marker; 1 = *L. plantarum* subsp. *plantarum* Kita-3; 2 = GJC2; 3 = LPK6; 4 = KLK5; 5 = DRK6; 6 = LVS1; 7 = GJS4; 8 = KLS4; 9 = LVC1; 10 = KLC3; 11 = DRS5; 12 = LPC1; M2 = 100 bp marker.

**Figure 3.**
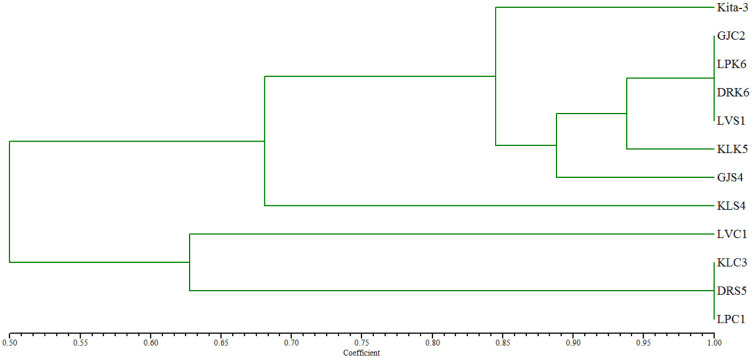
Dendrogram after rep-PCR using the BOXA1R primer for the 12 bacterial isolates.

Twelve bacterial isolates were identified using rep-PCR with the BOXA1R primer, including *L. plantarum* subsp. *plantarum* Kita-3 (Figure 2). The agarose gel electrophoresis results revealed that all of the isolates had repetitive DNA patterns that differed from *L. plantarum* subsp. *plantarum* Kita-3. The variations in the repetitive DNA patterns on the agarose gel indicated the different taxonomic levels [43]. The matrix data of the patterns were processed using NTSYSpc 2.1 software in Sequential Agglomerative Hierarchical Nesting, as carried out in previous studies to obtain the dendrogram [45]. The genetic relationships of the 12 isolates are shown in Figure 3. Based on the dendrogram, the isolates had similarity levels between 50% and 100%. Isolates with high similarity in the dendrogram have a relatively close genetic relationship. The high diversity of the dendrogram indicated that the method used had high sensitivity to distinguish isolates. When the homology rate reached 93%–95%, rep-PCR was applied as a genotyping technique [44],[46].

### 3.6. Bacterial identification based on 16S rRNA gene sequencing

**Figure 4.**
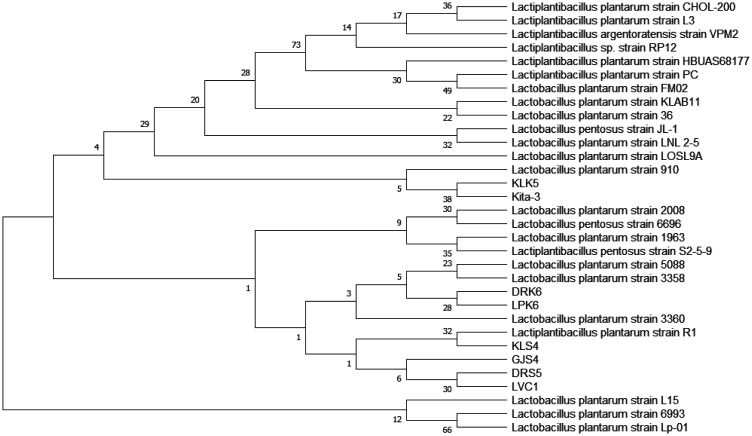
The genetic relationships between the sample isolates and the reference bacterial species in the NCBI's GenBank are based on 16S rRNA gene sequencing. The numbers in the phylogenetic tree indicate the percentage of bootstrap values from 1,000 repetitions.

The rep-PCR results were further processed by 16S rRNA gene sequencing. The bacterial isolates in the samples and the reference bacterial species from the NCBI's GenBank were examined for their genetic relationships using MEGA XI software and a phylogenetic tree [26]. Based on this result, *L. plantarum* subsp. *plantarum* Kita-3 had a distant genetic relationship with the other isolates from the blood and organs of the rats and was close to the isolate from the colon (Figure 4). The phylogenetic tree showed that the KLK5 isolate was located in the same branch as *L. plantarum* subsp. *plantarum* Kita-3 in the 16S rRNA gene sequencing. The KLK5 isolate obtained from the colon was indicated as a bacterium due to the consumption of *L. plantarum* subsp. *plantarum* Kita-3. Thus, *L. plantarum* subsp. *plantarum* Kita-3 was able to grow and survive in the gastrointestinal tract and adhered to the intestinal mucosa as a probiotic requirement. The DRK6, LPK6, KLS4, GJS4, DRS5, and LVC1 isolates were located in different branches of *L. plantarum* subsp. *plantarum* Kita-3. This indicated that the bacterial isolates in the blood and organs of the rats were not *L. plantarum* subsp. *plantarum* Kita-3, but indigenous bacteria that were growing in the rats since the beginning of the study. In addition, there were non-*L. plantarum* bacteria that grew on LPSM as a differential selective medium. This was due to the ability of several LAB to use sorbitol as an energy source and produce lactic acid from it [36]. It was confirmed that the bacteria detected in the rats' blood and organs were different from *L. plantarum* subsp. *plantarum* Kita-3 and were not the result of consuming a high dose of this strain. Therefore, *L. plantarum* subsp. *plantarum* Kita-3 did not translocate to the blood and organs of the rats.

## 4. Conclusions

This study showed that consumption of a high dose (10^11^ CFU/mL) of *L. plantarum* subsp. *plantarum* Kita-3 for 28 days by Sprague–Dawley rats had no adverse effects, including feed intake, body or organ weight, hematological parameters, biochemical markers, or gut morphology. *L. plantarum* subsp. *plantarum* Kita-3 survived and adhered in the rats' gastrointestinal tract, causing a significant increase in the *L. plantarum* population in the digesta, feces, and colon of the treated rats. We concluded that consuming a high dose of *L. plantarum* subsp. *plantarum* Kita-3 for 28 days did not trigger the translocation of bacteria in the blood or organs of the rats. Despite that this study was restricted to an animal study, the findings helped assess the safety of using *L. plantarum* subsp. *plantarum* Kita-3 as a probiotic.
